# Mutation and expression analysis in medulloblastoma yields prognostic variants and a putative mechanism of disease for i17q tumors

**DOI:** 10.1186/s40478-014-0074-1

**Published:** 2014-07-17

**Authors:** Gabriel A Bien-Willner, Robi D Mitra

**Affiliations:** Department of Pathology and Immunology, Washington University, Box 8118, 660 S, Euclid Ave, St. Louis, MO 63110 USA; Deptartment of Genetics, Washington University, St. Louis, MO USA

**Keywords:** Medulloblastoma, Group 4, i17q, Expression, Outcomes, Fluidigm, Next-generation sequencing

## Abstract

Current consensus identifies four molecular subtypes of medulloblastoma (MB): WNT, sonic hedgehog (SHH), and groups “3/C” and “4/D”. Group 4 is not well characterized, but harbors the most frequently observed chromosomal abnormality in MB, i17q, whose presence may confer a worse outcome. Recent publications have identified mutations in chromatin remodeling genes that may be overrepresented in this group, suggesting a biological role for these genes in i17q. This work seeks to explore the pathology that underlies i17q in MB. Specifically, we examine the prognostic significance of the previously-identified gene mutations in an independent set of MBs as well as to examine biological relevance of these genes and related pathways by gene expression profiling. The previously-implicated p53 signaling pathway is also examined as a putative driver of i17q tumor oncogenesis. The data show gene mutations associated with i17q tumors in previous studies (*KMD6A*, *ZMYM3, MLL3* and *GPS2*) were correlated with significantly worse outcomes despite not being specific to i17q in this set. Expression of these genes did not appear to underlie the biology of the molecular variants. *TP53* expression was significantly reduced in i17q/group 4 tumors; this could not be accounted for by dosage effects alone. Expression of regulators and mediators of p53 signaling were significantly altered in i17q tumors. Our findings support that chromatin remodeling gene mutations are associated with significantly worse outcomes in MB but cannot explain outcomes or pathogenesis of i17q tumors. However, expression analyses of the p53 signaling pathway shows alterations in i17q tumors that cannot be explained by dosage effects and is strongly suggestive of an oncogenic role.

**Electronic supplementary material:**

The online version of this article (doi:10.1186/s40478-014-0074-1) contains supplementary material, which is available to authorized users.

## Introduction

Medulloblastoma (MB) is the most common malignant brain tumor in children [1,2]. Risk stratification strategies that place patients into standard-risk (SR) and high-risk (HR) groups show 5-year survival rates of roughly 86(+/−9)% and 40% respectively [3-5]. However, clinical staging does not always accurately predict tumor behavior in the individual patient. Current treatment strategies, while often curative, frequently result in significant lifelong debilitation, and the limited predictive power of the two-tier stratification system likely significantly contributes to overall patient morbidity [6].

In an attempt to more accurately stratify patients, efforts have utilized the molecular genetics and genomics of these tumors to identify both prognostic markers and biological processes that may underlie the cause or progression of disease. Both the sonic hedgehog (SHH) and WNT signaling pathways have been known to be involved in subsets of MBs [7-9]. Other relevant prognostic markers in MB include anaplastic/large cell histology and *MYC* amplification, although these are of limited clinical utility due to their relative rarity [10-12]. The most common genetic abnormality seen in MB is i17q (or more specifically, idic(17)(p11.2)), a chromosomal rearrangement created by non-allelic homologous recombination at 17p11.2 that is relatively specific to MB in brain tumors [13-18]. Patients with this abnormality have shown to have early recurrence and worse outcomes in several studies [8,14,19-24]. Interestingly, this rearrangement is much more frequent in male patients. Due to the nature of this rearrangement, tumors with i17q are hemizygous for chromosome 17p telomeric to 17p11.2 while the remainder of the chromosome is duplicated. To date no specific mechanism has been proposed to explain pathogenesis of these tumors, although hemizygosity of genes and tumor suppressors on 17p such as *TP53* have been suspected. Anticipated “second-hit” *TP53* mutations have not been described in i17q-positive MBs and these hypotheses have been largely overshadowed recently by genomics and expression profiling of tumors [25-27]. However, these initial studies identified other p53 signal modifiers such as WIP1 that have not been completely explored and warrant further study.

Recent attempts to sub-classify tumors based on microarray gene expression profiling have led to a consensus that there are likely four MB molecular variants [28,29]. These studies have supported the previously described SHH and WNT variants, and have split remaining cases into “group 3/C” and “group 4/D”. Currently it is thought that group 3 tumors are driven by MYC expression and may have worse outcomes [28,30,31]. However, to date there has been relatively little molecular investigation into the pathogenesis of group 4, the most common subtype. Depending on the data sets used for the hierarchical clustering analysis of the expression data, i17q cases fall either primarily into group 4 (with the remaining cases falling into group 3) or entirely within group 4, while making up a majority of its cases. This suggests that this chromosomal aberration may be critical to the pathogenesis of these tumors. A recent cytogenetic study of over 1000 tumors identifies i17q in ¾ of all group 4 tumors [24]. Other rare chromosomal rearrangements such as MYCN/CDK6 amplifications and more recently SNCAIP duplication have also been associated with group 4 tumors [29,32].

Attempts to identify MB variant-specific mutations have been recently undertaken with both whole-genome and exome sequencing in two large MB sample sets to further define the disease [33,34]. These studies identified several mutations that were overrepresented or exclusive to MB variants, including those harboring i17q. Many of the identified mutations in i17q tumors are in chromatin remodeling genes, including histone methyltransferases (*MLL3*), histone demethylases (*KDM6A*), and histone de-acetylases (*ZMYM3* and *GPS2*), suggesting that these pathways may be key to their pathogenesis.

In this work we explore the specificity and prognostic significance of mutations in the previously identified (i17q-associated) chromatin remodeling genes in an independent cohort of 57 MBs. The expression of genes in pathways involved in both chromatin remodeling and p53 signaling are also examined in 103 medulloblastomas to see if these correlate with i17q status or the established molecular MB variants, and may thus underlie the biology or oncogenesis of these tumors.

## Materials and methods

### Patients

Patients for gene sequencing were selected based on a diagnosis of medulloblastoma from the archives of WUSM in accordance with an approved institutional review board protocol (# 201104083) (Washington University HRPO) and their details have been previously published [19]. SR patients were identified by a documented lack of residual disease as well as a lack of drop metastases in brain and spinal magnetic resonance imaging (MRI) status post-surgical resection, as well as negative CSF cytology. HR patients typically had either residual disease or drop metastases (as reported in post-surgical or radiological reports). Patients with *MYC* amplifications were also placed in the high-risk group. Patient age was not used as an identifier of high risk given conflicting literature [35-37]. The data used for expression analyses are publicly available and the details of those patients, as well as the methods of expression profiling, have been published elsewhere [28]. A table summarizing clinical and molecular information for each patient can be found in Additional file 1: Table S1.

### Sample preparation

Formalin-fixed, paraffin embedded (FFPE) blocks of patient tumors and control (non-tumor) material were evaluated for tumor cell content by a pathologist and cored (G.A.B.). Multiple 2 mm cores of both tumor and control tissue were obtained per case when available. Tumor cores were homogeneous with tumor comprising >90% of the sampled area. DNA was extracted with the Gentra Purgene Kit (Qiagen, Valencia, CA). Samples were treated with proteinase K until all tissue was digested (up to four days). DNA yields ranged from 1–3100 μg (mean 593 $\mu$g, median 218 μg; 93% of samples > 10 μg). For sequencing, tumor samples were prepared at a concentration of ~250 ng/μl.

### Gene amplification and sequencing

Genes of interest (*MLL3, GPS2, KDM6A,* and *ZMYM3*) were selected based on whole-genome and exome-based studies as being specific to, or over-represented in i17q + MB samples and having a role in chromatin remodeling [33,34]. Amplification of the coding regions was undertaken with the Fluidigm microfluidics-based Access Array system (San Francisco, CA). Primer sets were designed by Fluidigm to cover these regions as 192 unique amplicons (see Additional file 2: Table S2). After sample target amplification and barcoding, samples were sequenced with the Illumina MiSeq 2 × 250 platform (San Diego, CA). The mean and median percent of amplicons per sample in the data set passing quality and coverage thresholds of 30 reads/base were 84% and 92%, respectively. Average and median depth of coverage across all amplicons was 4795 and 1646 reads/base, respectively.

### Data analysis

DNA alignments were performed with the Novoalign software (Novocraft, Selangor, Malaysia). Variant calls were made with the FreeBayes software package, with minimum quality and mapping scores of 30 and a minimum alternate allele fraction of 0.20 [38]. Annotation was performed with the ANNOVAR software package [39]. Variants were filtered to remove putative synonymous or intronic (non-splice site) changes, as well as polymorphisms documented as >1% prevalence in the dbSNP, 1000 genomes, or 6500 exomes databases (located at http://www.ncbi.nlm.nih.gov/projects/SNP/, http://www.1000genomes.org/, http://evs.gs.washington.edu/EVS/ respectively) [40,41]. Because amplification-based methods result in a high number of false positives in FFPE samples due to crosslinking and deamination events [42], stringent variant calling conditions were used to maximize specificity. First, microfluidics based amplification and sequencing was repeated for all samples (at least two times) as recommended by recent publications [43]. This would serve to validate any variant calls as well as to increase depth of coverage across a maximum number of amplicons. Secondly, variant calling was conducted across compiled BAM files from multiple runs to prevent variant calling of “jackpotting” events from entering downstream data analyses. Finally, variants were only considered if they were identified with the variant caller software and present in a replicate at a frequency of >5% as identified by the Integrated Genome Viewer [44]. This approach was verified by Sanger sequencing 17 putative mutations or low frequency polymorphisms that met these criteria, as well as Sanger sequencing 154 variants that were identified but failed to meet this criteria (variants that were called on a single replicate). This filter technique yielded a specificity and sensitivity of 100%.

All statistical analyses were performed with the R statistical software and the survival statistical package [45,46]. Kaplan-Meier survival curve significance was measured with the log-rank test.

## Results

### Mutations in chromatin remodeling genes are not limited to i17q positive tumors and seen in the SHH group

Mutations in genes over-represented in i17q tumors may underscore the biology of the disease by identifying critical pathways for tumorigenesis and may also explain the adverse outcomes observed in these patients. To explore this, the four previously-identified genes involved in chromatin remodeling associated with i17q (*KDM6A, ZMYM3, MLL3*, and *GPS2*) were sequenced in an independent cohort of 57 consecutive medulloblastomas. Mutations were identified in *KDM6A GPS2*, and *MLL3* (Table 1). 13 mutations were identified in 10 (18%) patients, more than expected based on the previous reports. 5/10 (50%) patients with mutations were identified in the standard-risk group. A majority of the mutations were seen in *MLL3* (85%). Missense mutations were a majority of the variant calls (69%). These variants were assessed for their putative damage to protein function with AV_SIFT, PolyPhen, LRT, and the MutationTaser computer programs. All of the variants identified were predicted to be damaging by at least one program (see Additional file 3: Table S3). Mutations were identified in patients with and without i17q, as well as in patients in the SHH variant. Three of the 10 patients (30%) were in the SHH group. Only one of the variants identified was in a patient with idic(17)(p11.2). Interestingly, these mutations were seen in 2/4 patients in this data set with rearrangements outside the REPA/REPB region (#32 and #28). These data suggest that in this cohort of patients, mutations in these genes are not specific to i17q and thus cannot explain their biology or outcomes.

**Table 1:** **Mutations identified in 57 consecutive medulloblastoma cases**

**Sample**	**Location**	**Gene**	**Exonic function**	**Damaging**	**Chr**	**Start**	**Ref**	**Obs**
32	Exonic; splicing	GPS2	Splice site	--	chr17	7217225	C	T
41	Exonic; splicing	KDM6A	Frameshift insertion	--	chrX	44969494	-	GG
17	Exonic	MLL3	Nonsynonymous	4/4	chr7	151859899	G	A
16	Exonic	MLL3	Nonsynonymous	2/4	chr7	151860230	G	C
16	Exonic	MLL3	Nonsynonymous	1/4	chr7	151877127	G	T
21	Exonic	MLL3	Nonsynonymous	4/4	chr7	151879265	G	T
21	Exonic	MLL3	Stopgain	--	chr7	151900023	A	T
13	Exonic	MLL3	Stopgain	--	chr7	151874686	G	A
21	Exonic	MLL3	Nonsynonymous	3/4	chr7	151875073	G	A
30	Exonic	MLL3	Nonsynonymous	3/3	chr7	151927021	C	A
28	Exonic	MLL3	Nonsynonymous	1/4	chr7	151919690	C	T
33	Exonic	MLL3	Nonsynonymous	3/4	chr7	151970877	G	A
20	Exonic	MLL3	Nonsynonymous	2/4	chr7	151945568	C	T

“Damaging” is the number of mutation analysis programs (out of four) that predict altered protein function as a result of the variant. Please note that these programs do not make predictions for stopgain, frameshift, or splice site change mutations.

### Mutations in MLL3 and other chromatin remodeling genes are associated with recurrence and worse overall survival

**Figure 1:**
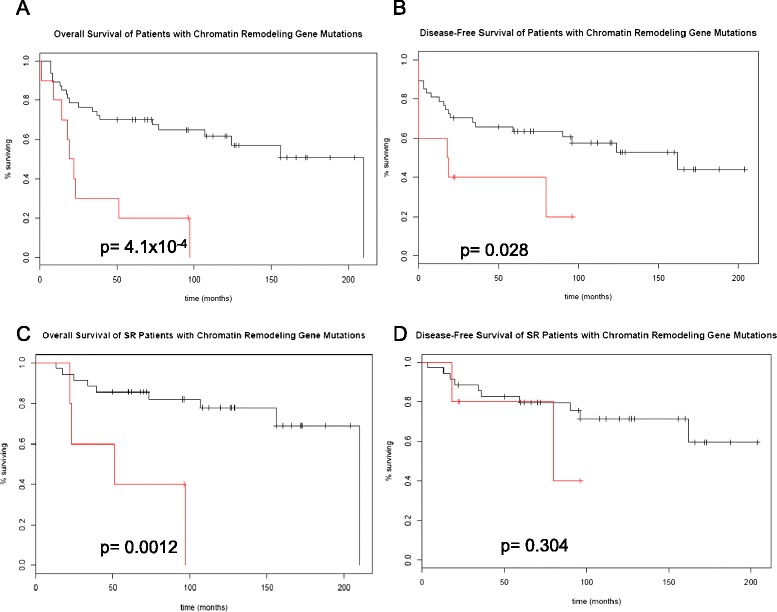
**Kaplan-Meier survival curves of 57 patients with and without mutations in chromatin remodeling genes.** P-values are calculated with the log-rank test. The red plot indicates survival of patients with mutations in *MLL3/KMT2C, KDM6A,* or *GPS2*. Mutations were identified in a pipeline that consisted of the amplification-based Fluidigm Access Array system, massively-parallel sequencing of the exons of select genes with the Illumina MiSeq (2x250), and post-sequencing variant detection followed by annotation with the FreeBayes and ANNOVAR software packages, respectively. **A.** Overall survival (OS) measured in the standard-risk (SR) group. **B.** OS across all patients. **C.** Disease-free survival (DFS) in the SR group. **D.** DFS in all patients.

**Figure 2:**
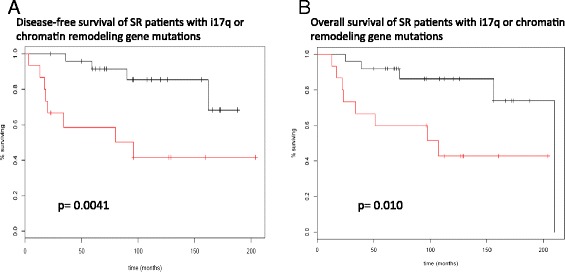
**Kaplan-Meier survival curves combining molecular risk factors shown to be significant in the cohort of 57 patients.** The red survival plot includes patients who had either mutations in chromatin remodeling genes or i17q. **A.** DFS in the SR group. **B.** OS in the SR group.

Although not specific to i17q samples, chromatin remodeling gene mutations were correlated with outcomes with Kaplan-Meier survival analyses. Patients with mutations in *MLL3, KDM6A*, and *GPS2* had worse outcomes in terms of overall survival (OS) and disease-free survival (DFS) than those without mutations (Figure 1). Interestingly, combining prognostic factors shown to be significant in this cohort (i17q status and mutations in chromatin remodeling genes) resulted in a powerful predictive tool within this data set. This approach identifies all but one of the patients in the SR group that recur and all but two patient deaths at 5 years (p = 0.0041 and p = 0.010, respectively (Figure 2). Similar findings were seen with OS and DFS across all patients (p = 0.033 and p = 0.042, respectively). Combined with i17q status, the identification of these mutations showed significant associations with poor outcomes in terms of OS and DFS.

### Expression of chromatin remodeling genes identified by mutation profiling

**Figure 3:**
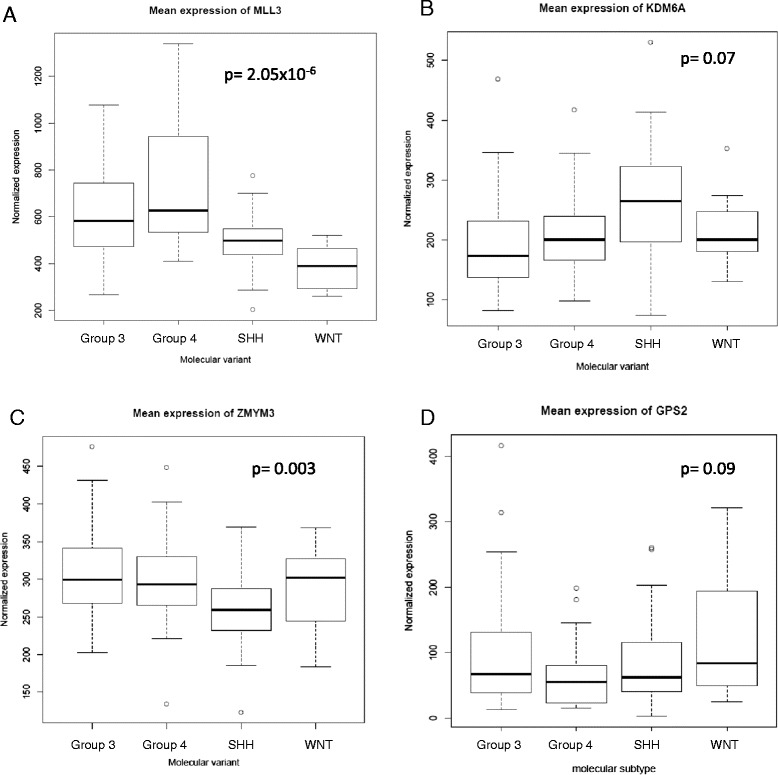
**Expression of chromatin remodeling genes identified as being over-represented in i17q in prior studies.** Boxplots indicate expression levels as identified in expression microarrays for 103 MBs for each molecular variant. The data are normalized based on the number of probes per feature, allowing for direct comparisons of expression across different genes. Black lines indicate the mean values while the box includes data within one standard deviation. P values are calculated as variance from the grand mean of all MB, or ANOVA (one way). **A.** Expression of *MLL3* in the four molecular MB variants. *MLL3* is significantly differentially expressed across the molecular variants with groups 3 and 4 having higher levels, unlike the other genes identified. **B,** **C,** **D.** Expression levels of *KDM6A*, *ZMYM3*, and *GPS2* across the four MB variants. There is no significant difference in expression of these genes from the grand mean that correlate with i17q-associated variants.

The four distinct molecular variants of MB, as they are currently understood, are classified by their differing gene expression profiles. It stands to reason that mutations that are subtype-specific may uncover pathways that when altered are essential for tumor pathogenesis. If so, the expression of these genes and pathways would be expected to also be subtype-specific. Chromatin remodeling genes identified by mutational analyses were compared for differences in expression across the four molecular MB subtypes as well as in the presence or absence of i17q in 103 MB cases using an Affymetrix exon array platform. This cohort includes 8 WNT, 33 SHH, 27 group 3, and 35 group 4 cases. As expected based on the mutation profiling in our cohort, there was no clear association between molecular variants and expression of the four chromatin remodeling genes of interest. A small but significant difference in expression across molecular subtypes was observed for the histone methyltransferase-coding *MLL3* (p = 2.05 × 10^−6^, ANOVA), whose mean expression was higher in groups 3 and 4, and for *ZMYM3*, which had a lower expression in the SHH group, although the magnitude of change is not large for either gene (p = 0.003) (Figure 3). The presence of i17q may be indicative of a unique biological entity; however, similar to what was observed across the different molecular subtypes there was no clear association between expression of the identified chromatin remodeling genes and i17q status. Other than *GPS2*, none of the identified genes were differentially expressed in i17q-positive tumors (see Additional file 4: Figure S1). The fact that *GPS2* was seen in lower levels in i17q tumors is not surprising given its location (17p13), and this finding is likely explained by dosage effect due to hemyzygosity of 17p in these cases. While KDM6A levels are not different between molecular variants or i17q positive and negative tumors, it must be added that differences between variants were observed in EZH2, a methyltransferase that acts opposite to KDM6A function (see Additional file 4: Figure S2) [47]. These findings suggest that the chromatin remodeling genes identified by mutational analyses as subtype- or i17q-specific are not likely critical for pathogenic differences seen between MB variants, although the role of other such genes may be relevant to these processes.

### Expression of other chromatin remodeling genes can be variant-specific

Although the expression of chromatin remodeling genes identified by mutational studies did not seem to be specific to molecular subtypes, it is still possible that chromatin remodeling genes or other epigenetic phenomena play an essential role in the development of these tumors. Other genes involved in histone modification were assessed for their relative expression across the different MB variants to test if there was any specificity to their expression and thus could be critical to their development. The genes of interested included member of the *HDAC* gene family as well as other chromatin remodeling genes associated with MB specificity (Additional file 4: Figure S2 and Additional file 4: Figure S3). The *HDAC* genes often had marked differences in expression amongst the different MB subtypes (Additional file 4: Figure S2). *HDAC1* expression is significantly decreased in group 4 MBs, while *HDAC4* expression is significantly decreased in group 3. Also identified as a common target for mutation in MB, *MLL2,* showed differential expression with significantly lower expression in the SHH group. This is particularly interesting because mutations in this gene are also observed in this group [34]. Other selected chromatin remodeling associated genes that are differentially expressed among the MB variants can be seen Additional file 4: Figure S3. The epigenetic difference across the molecular subtypes does not necessarily end with histone modification. DNA methyltransferase expression profiles also show clear differences among the different MB variants, where the SHH tumors have significantly lower expression of *DNMT1* and *DNMT3A* (Additional file 5: Figure S4). These data suggest that differences in gene expression of chromatin remodeling and other epigenetic pathways appear to be profound in MB variants, but it is unclear if these changes reflect variant-specific noise or if they may underlie the biology of disease. Furthermore, these data hint at possible new targets for differentiating group 3 and 4 tumors, where existing antibody targets have not proven reliable [19,20].

### Expression of TP53 pathway genes in i17q-positive tumors

**Figure 4:**
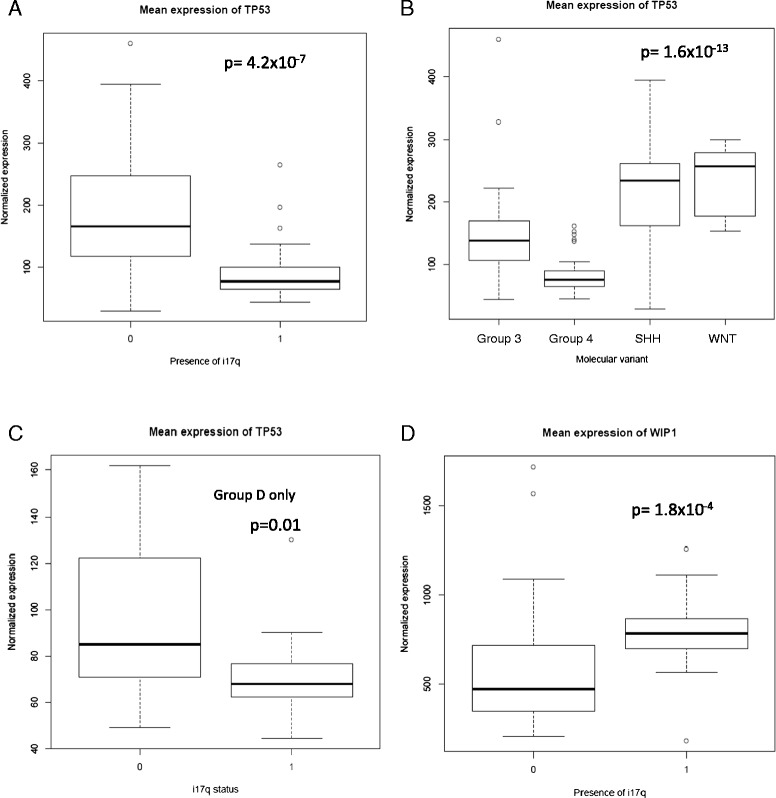
**Expression of** ***TP53*** **and related genes in i17q and across the MB variants. A.** Mean expression of TP53 in i17q-positive and negative patients. “1” indicates i17q-positive status. There was significantly lower expression (p = 4.2 × 10^−7^, t-test) in i17q-positive tumors. **B.** TP53 expression was similarly decreased across group 4 tumors, which contain a majority of i17q-positive cases. **C.** When comparing only within group 4 cases, tumors with i17q express significantly lower levels of *TP53*. This suggests that the presence of i17q is a significant cause of lower *TP53* expression. **D.** *WIP1* is overexpressed in i17q-positive cases and functions to suppress p53 signaling on both a DNA and protein level, further altering this signaling pathway in these tumors.

Tumor suppressor p53 has previously been implicated as a driver mutation in i17q tumors as has as a negative regulator of p53 signaling, WIP1, whose function is to inhibit *TP53* expression and to promote p53 degradation [48,49]. The structure of i17q is particularly intriguing in that its formation causes both hemizygosity of *TP53* and duplication of *WIP1* (located on chr17q), hinting that this may be a tumor-initiation event in this subset of MB. To test if *TP53* or *WIP1* were aberrantly expressed in i17q MB, and if these differences were subtype-specific, 103 MB tumors were examined for the expression p53-related genes in i17q-positive and negative samples (Figure 4). *TP53* expression is significantly decreased in i17q tumors (p = 4.2 × 10^−7^). A significant decrease in *TP53* expression is seen across group 4 tumors when compared to other variants (p = 1.6 × 10^−13^, ANOVA, Figure 4B), a difference that is not as pronounced as in i17q tumors but also not accounted for by the presence of i17q tumors within this group (Figure 4D; Additional file 5: Figure S5). This may demonstrate the similar biology underscoring i17q and the remaining group 4 tumors. As expected due to dosage effects, *WIP1* is over-expressed in i17q-positive tumors compared to non-17q tumors (p = 1.8 × 10^−4^) [25]. *WIP1* is also overexpressed in group 4 tumors compared to other MB variants (Additional file 5: Figure S5). These data support that key regulators of the p53 signaling pathway are aberrantly expressed in a MB-variant/i17q-specific manner.

**Figure 5:**
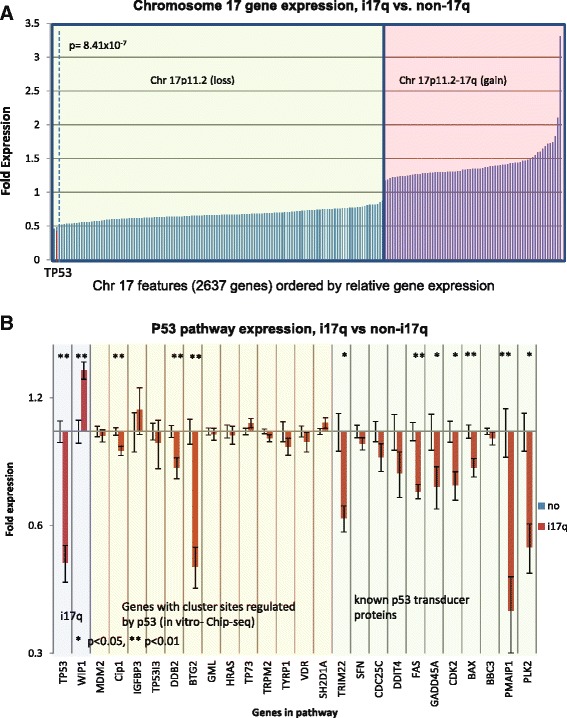
**i17q tumors have significantly altered p53 signaling, and lowered levels of** ***TP53*** **expression cannot be explained by dosage effects alone. A.** Relative expression across all chromosome 17 genes for i17q cases. All features in the 103 MB expression microarray across chromosome 17 in i17q-positive tumors were normalized by negative cases and plotted by fold-expression on the X-axis. The green-shaded box contains all the genes in the hemizygous region telomeric to 17p11.2, while the red-shaded box contains all features centromeric to 17p11.2 and 17q. Although the latter contains more DNA, it is represented by fewer features on the microarray chip. The green box shows that there is a very broad distribution of expression of genes in the hemizygous region, and suggests very uniform lower levels of expression caused by dosage effects. With bonferroni correction, *TP53* is significantly reduced in i17q patients (p = 8.41 × 10^−7^, t-test). More so, it is expressed at levels greater than 2 standard-deviations less than the average gene in the hemizygous region across all i17q-positive patients, as represented by the red bar. The dotted line represents 2 standard deviations from the mean expression across the hemizygous region of i17q patients. **B.** Relative expression of genes in the p53 signaling pathway in i17q patients. Fold expression of genes thought to be related directly or indirectly to p53 signaling by chIP-seq experiments or the literature were measured. The blue box contains *TP53* and *WIP1*. The yellow box contains genes identified by chIP-seq experiments to be targets of p53 [51]. The green box contains genes understood to be p53 signal transducers in the literature. A single asterisk represents a p < 0.05 with Bonferroni correction and two asterisks represent p < 0.01. These data demonstrate a significant dysregulation of a majority of p53-related genes in i17q tumors.

Because *TP53* is located on 17p it could be assumed that the differences in expression between i17q positive and negative tumors are due to dosage effects alone, and thus the differences of expression could be a “passenger effect” of the tumor despite the fact that i17q-negative tumors are also likely suppressing *TP53* via mutation, epigenetics, or other means [26,50]. Alternately, robust changes in p53 signaling, as well as alterations in *TP53* expression beyond that expected by position effects alone suggests that p53 alterations are not a mere reflection of changes in chromatin content. Specifically, two hypotheses arise: first, that *TP53* expression is impacted by the same dosage effects that affect all of 17p genes in i17q as a result of hemizygosity; and second, that the unique formation of idic(17)(p11.2) suppresses p53 signaling beyond that which is expected by dosage effects alone and could be an oncogenic event. If the first hypothesis is correct, expression of *TP53* should behave similarly to other genes on 17p when comparing a ratio of expression in i17q to non-i17 cases. The alternate hypothesis predicts *TP53* expression to be significantly lower than other genes within 17p in the same scenario. To test whether *TP53* expression is more dysregulated than expected by position effects alone in i17q cases, expression of all gene positions in i17q were normalized by non-i17q tumor samples (2637 features) and the expression of *TP53* was compared to other similarly hemizygous genes in i17q tumors. This analysis shows that *TP53* expression is significantly decreased compared to all other genes in 17p affected by the same dosage effects (corrected p = 8.4 × 10^−7^) (Figure 5A). Furthermore, several genes that are either targets or mediators of p53 as established in the literature or found via chIP-seq experiments are significantly altered in i17q-positive tumors (Figure 5B) [51]. These data strongly suggest that i17q tumors have a unique alteration of the p53 signaling pathway that is known to be tumorigenic and could underlie the pathogenesis of this disease. However, such observations would need to be seen on other patient cohorts for validation and be supported by functional studies.

### Sex-specific nature of i17q suggests hormonal role in pathogenesis

Yet to be thoroughly explored is the reason for the male bias in i17q cases. In three studies alone [19,28,33] boys comprised 31/37, 16/18, and 10/13 i17q-positive MBs (~84%). One possible explanation is sex hormonal differences between boy and girls and their impact on p53 signaling. Recent studies have suggested that prepubescent girls may have significantly higher levels of estradiol than boys [52]. If true, this could be of significance as such hormones have complex regulatory interactions with p53 signaling. Specifically, data have demonstrated that estradiol increases p53 levels in a time and dose-dependent manner [53]. The estrogen-related receptor γ (ERRγ) has been shown to be able to repress estradiol-induced estrogen response elements, thereby inhibiting the effects of estradiol in certain conditions [54]. *ESRRG,* which codes for ERRγ, is expressed at significantly higher levels in i17q-positive and group 4 tumors in our data set; differences that cannot be accounted for by sex-specific expression (t-test, p = 1.5 × 10^−5^) (Additional file 5: Figure S6). While these findings are highly speculative, it is possible that circulating estradiol in females is often sufficient to prevent tumors with the formation of i17q by maintaining a minimum threshold level of p53 activity.

## Discussion

Group 4 is the most common variant of MB and includes the most common genetic abnormality, yet little is known about the pathogenesis of these tumors. The data presented supports the idea that the formation of idic(17)(p11.2) may be sufficient to cause oncogenesis. We hypothesize that the REPA/REPB-mediated formation of idic(17)(p11.2) does two things; first, it creates hemzigosity of 17p lowering p53 levels due to dosage effects; and second, the duplication of *WIP1* (and potentially other factors) further suppresses *TP53* expression and thus p53 signaling to levels that render it ineffective. This may explain why *TP53* mutations are not observed in i17q-positive tumors- there may be no selective advantage to additional loss in p53 when it is already markedly reduced in these cases. How chromatin remodeling may affect this process is not immediately clear.

The chromatin remodeling gene mutations tested were not specific to i17q tumors in this cohort and there was no evidence that these genes were important to the pathogenesis of disease. Admittedly, however, no *ZMYM3* mutations were identified, and only single *KDM6A* and *GPS2* variants were observed, so it is possible that in a larger sample set these mutations would have shown a similar distribution to other reports. Other limitations of this study constrain our analyses. The lack of available germline material prohibits absolute certainty that the variant calls are not rare polymorphisms absent from the established databases. Similarly, the nature of the Fluidigm platform limits our ability to interpret allelic ratios and is more likely to yield false-positive and false-negative calls in this setting compared to other methods. Regarless, the number of *MLL3* variants observed were more than expected based on the results of the works of Pugh or Robinson et al. [33], and were similar to the number of mutations identified in Parsons et al. [55], suggesting that either there is still a heterogeneity of samples available in the literature or that genomic approaches of variant detection require further refinement, particularly in the arduous task of variant filtering and validation [33,34,55]. Patients with mutations in these genes did have worse outcomes, in terms of disease-free survival and overall survival. This is consistent with other tumors that have similarly shown that mutations in chromatin remodeling genes purport worse patient outcomes [56]. It is possible that aggressive tumors are acquiring mutations and epigenetic changes that help promote tumor growth, and mutations in chromatin remodeling genes may simply be a reflection of this process as it may have global effects on gene expression. The differences in *HDAC* or *DNMT* genes may reflect the molecular and cellular origins of the tumors or may reflect the pathogenesis of disease. Further studies on these pathways are warranted to address such questions.

The analyses provided in this cohort of 57 MB cases show that patients with mutations in the chromatin remodeling genes *MLL3, GPS2*, and *KDM6A* have significantly worse outcomes in terms of DFS and OS. Combining these results with previously published data on these cases regarding i17q status identifies a majority of all recurrences in this data set. These data suggests that there may be clinical utility in screening SR patients for both i17q status and chromatin remodeling gene mutations, particularly *MLL3*. Furthermore, these data demonstrate that cytogenetic and molecular studies could be readily implemented for more specific patient stratification and prognostication without the need for expression profiling of cases; a labor that is impractical in the current laboratory setting and currently has no validated alternatives for the separation of group 3 and 4 tumors [19,20]. Screening for these mutations in other MB cohorts is warranted to validate these findings, and the results may ultimately add to the growing literature of molecular markers shown to be useful in characterizing and stratifying MB patients.

## Additional files

Additional file 1: Table S1. Clinical and molecular information for the 57 MB samples. A “1” designates the presence of a characteristic and a “0” signifies its absence. Additional file 2: Table S2. Location and name of the amplicons used for the Fluidigm platform and subsequent sequencing. Additional file 3: Table S3.
*In silico* analyses of amino acid changes with different predictive software. Allelic ratios reflect the range of ratios observed in the different Fluidigm runs. The variance is due to the nature of this platform being amplification based, and the frequency of “jackpotting” events. Great caution should be taken when attempting to derive meaning from these ratios. Additional file 4: Figure S1. Expression of differentially-expressed chromatin remodeling genes in the four MB variants. Differential expression (mean values) was seen in histone deacetlyases, histone demethylases, histone methyltransferases, and other chromatin remodelers. These data suggest that, in general, group 3 and 4 tumors tend to increase histone methyltransferase activity while suppressing demethylase activity. Histone deacetylases vary tremendously by MB variant. **Figure S2.** Expression of chromatin remodeling genes differentially expressed across the four MB variants or previously associated with differential expression. Histone deacetylase genes *HDAC1 and HDAC2* show differential expression across the MB variants, with decreased expression in groups D and C respectively. Histone methyltransferase-coding *MLL2* is decreased in SHH MB, and is also frequently mutated in that group. EZH2 is involved in the H3K27 trimethylation, which is removed by KDM6A. Its gene *EZH2* is relatively over-expressed in group 3 and 4 tumors. Previous studies had suggested that *UTY* (a paralog of *KDM6A*) and *CDH7* may be decreased in expression in group 3 and 4 tumors in an effort to maintain an epigenetic stem-like state [40]. Significant differences for expression in these groups were not seen in this data set; however, this does not imply a stem-like epigenetic state is not maintained through other means in these tumors. **Figure S3.** Expression of chromatin remodeling genes whose mutations are over-represented in group 4/i17q in patients with and without i17q. Other than *GPS2*, there is no statistical significance (student t-test, 2-tailed) in expression of these genes between i17q-positive and negative tumors. The difference in *GPS2* is likely explained by dosage effect as it is positioned in chromosome 17p. Additional file 5: Figure S4. Expression of DNA-methyltransferases is differentially expressed across MB variants. The SHH variant shows lower levels of expression for both *DNMT1* and *DNMT3A*. **Figure S5.** Expression of *TP53* and *WIP1* in the molecular variants of MB. The top panel shows expression of *TP53* in the MB molecular variants independent of any i17q-positive cases. Group 4 shows significantly lower expression, although it is not as low as seen in in i17q + tumors. The bottom figure shows that WIP1 is also differentially expressed among the MB variants, with significantly higher expression in Group 4. **Figure S6.** *ESRRG* expression in MB variants, i17q, and male and female sexes. ERRγ is an estrogen receptor that has no known ligand but can interfere with estradiol signaling. It has been shown than in ESRα-expressing cells, overexpression of ERRγ suppresses estradiol-mediated expression of its response elements. A. *ESRRG* codes for ERRγ and is significantly overexpressed in i17q tumors (p = 1.5 × 10^−5^, t-test). B. Similarly, group 4 tumors showed significantly higher levels compared to the other groups. C. Although male patients are significantly overrepresented in i17q-positive tumors and group 4 cases, male gender alone was not significantly associated with increased *ESRRG* expression, suggesting that this is intrinsic to group i17q/4 tumors.

## References

[1] Gurney JG, Smith MA, Bunin GR, Reis LAG, Smith MA, Gurney JG (1999). CNS and miscellaneous intracranial and intraspinal neoplasms. Cancer incidence and survival among children and adolescents: United States SEER program 1975–1995, vol NIH Pub. No. 99–4649.

[2] Gurney JG, Kadan-Lottick N (2001). Brain and other central nervous system tumors: rates, trends, and epidemiology. Curr Opin Oncol.

[3] Packer RJ, Gajjar A, Vezina G, Rorke-Adams L, Burger PC, Robertson PL, Bayer L, LaFond D, Donahue BR, Marymont MH, Muraszko K, Langston J, Sposto R (2006). Phase III study of craniospinal radiation therapy followed by adjuvant chemotherapy for newly diagnosed average-risk medulloblastoma. J Clin Oncol.

[4] Packer RJ, Sutton LN, Elterman R, Lange B, Goldwein J, Nicholson HS, Mulne L, Boyett J, D'Angio G, Wechsler-Jentzsch K, Reaman G, Cohen BH, Bruce DA, Rorke LB, Molloy P, Ryan J, LaFond D, Evans A, Schut L (1994). Outcome for children with medulloblastoma treated with radiation and cisplatin, CCNU, and vincristine chemotherapy. J Neurosurg.

[5] Gajjar A, Chintagumpala M, Ashley D, Kellie S, Kun LE, Merchant TE, Woo S, Wheeler G, Ahern V, Krasin MJ, Fouladi M, Broniscer A, Krance R, Hale GA, Stewart CF, Dauser R, Sanford RA, Fuller C, Lau C, Boyett JM, Wallace D, Gilbertson RJ (2006). Risk-adapted craniospinal radiotherapy followed by high-dose chemotherapy and stem-cell rescue in children with newly diagnosed medulloblastoma (St Jude Medulloblastoma-96): long-term results from a prospective, multicentre trial. Lancet Oncol.

[6] Polkinghorn WR, Tarbell NJ (2007). Medulloblastoma: tumorigenesis, current clinical paradigm, and efforts to improve risk stratification. Nat Clin Pract Oncol.

[7] Ellison DW, Kocak M, Dalton J, Megahed H, Lusher ME, Ryan SL, Zhao W, Nicholson SL, Taylor RE, Bailey S, Clifford SC (2010). Definition of disease-risk stratification groups in childhood medulloblastoma using combined clinical, pathologic, and molecular variables. J Clin Oncol.

[8] Pfister S, Remke M, Benner A, Mendrzyk F, Toedt G, Felsberg J, Wittmann A, Devens F, Gerber NU, Joos S, Kulozik A, Reifenberger G, Rutkowski S, Wiestler OD, Radlwimmer B, Scheurlen W, Lichter P, Korshunov A (2009). Outcome prediction in pediatric medulloblastoma based on DNA copy-number aberrations of chromosomes 6q and 17q and the MYC and MYCN loci. J Clin Oncol.

[9] Pomeroy SL, Tamayo P, Gaasenbeek M, Sturla LM, Angelo M, McLaughlin ME, Kim JY, Goumnerova LC, Black PM, Lau C, Allen JC, Zagzag D, Olson JM, Curran T, Wetmore C, Biegel JA, Poggio T, Mukherjee S, Rifkin R, Califano A, Stolovitzky G, Louis DN, Mesirov JP, Lander ES, Golub TR (2002). Prediction of central nervous system embryonal tumour outcome based on gene expression. Nature.

[10] Eberhart CG, Kepner JL, Goldthwaite PT, Kun LE, Duffner PK, Friedman HS, Strother DR, Burger PC (2002). Histopathologic grading of medulloblastomas: a pediatric oncology group study. Cancer.

[11] Perry A (2002). Medulloblastomas with favorable versus unfavorable histology: how many small blue cell tumor types are there in the brain?. Adv Anat Pathol.

[12] Brown HG, Kepner JL, Perlman EJ, Friedman HS, Strother DR, Duffner PK, Kun LE, Goldthwaite PT, Burger PC (2000). "Large cell/anaplastic" medulloblastomas: a pediatric oncology group study. J Neuropathol Exp Neurol.

[13] Bayani J, Zielenska M, Marrano P, Kwan Ng Y, Taylor MD, Jay V, Rutka JT, Squire JA (2000). Molecular cytogenetic analysis of medulloblastomas and supratentorial primitive neuroectodermal tumors by using conventional banding, comparative genomic hybridization, and spectral karyotyping. J Neurosurg.

[14] Nicholson J, Wickramasinghe C, Ross F, Crolla J, Ellison D (2000). Imbalances of chromosome 17 in medulloblastomas determined by comparative genomic hybridisation and fluorescence in situ hybridisation. Mol Pathol.

[15] McCabe MG, Ichimura K, Liu L, Plant K, Backlund LM, Pearson DM, Collins VP (2006). High-resolution array-based comparative genomic hybridization of medulloblastomas and supratentorial primitive neuroectodermal tumors. J Neuropathol Exp Neurol.

[16] Mendrzyk F, Korshunov A, Toedt G, Schwarz F, Korn B, Joos S, Hochhaus A, Schoch C, Lichter P, Radlwimmer B (2006). Isochromosome breakpoints on 17p in medulloblastoma are flanked by different classes of DNA sequence repeats. Genes Chromosomes Cancer.

[17] Carvalho CM, Lupski JR (2008). Copy number variation at the breakpoint region of isochromosome 17q. Genome Res.

[18] Shaw CJ, Lupski JR (2004). Implications of human genome architecture for rearrangement-based disorders: the genomic basis of disease. Hum Mol Genet.

[19] Bien-Willner GA, Lopez-Terrada D, Bhattacharjee MB, Patel KU, Stankiewicz P, Lupski JR, Pfeifer JD, Perry A (2012). Early recurrence in standard-risk medulloblastoma patients with the common idic(17)(p11.2) rearrangement. Neuro Oncol.

[20] Min HS, Lee JY, Kim SK, Park SH (2013). Genetic grouping of medulloblastomas by representative markers in pathologic diagnosis. Transl Oncol.

[21] Scheurlen WG, Schwabe GC, Joos S, Mollenhauer J, Sorensen N, Kuhl J (1998). Molecular analysis of childhood primitive neuroectodermal tumors defines markers associated with poor outcome. J Clin Oncol.

[22] Gilbertson R, Wickramasinghe C, Hernan R, Balaji V, Hunt D, Jones-Wallace D, Crolla J, Perry R, Lunec J, Pearson A, Ellison D (2001). Clinical and molecular stratification of disease risk in medulloblastoma. Br J Cancer.

[23] Cogen PH, McDonald JD (1996). Tumor suppressor genes and medulloblastoma. J Neurooncol.

[24] Shih DJ, Northcott PA, Remke M, Korshunov A, Ramaswamy V, Kool M, Luu B, Yao Y, Wang X, Dubuc AM, Garzia L, Peacock J, Mack SC, Wu X, Rolider A, Morrissy AS, Cavalli FM, Jones DT, Zitterbart K, Faria CC, Schuller U, Kren L, Kumabe T, Tominaga T, Shin Ra Y, Garami M, Hauser P, Chan JA, Robinson S, Bognar L et al (2014) Cytogenetic prognostication within medulloblastoma subgroups. J Clin Oncol doi:10.1200/JCO.2013.50.9539 10.1200/JCO.2013.50.9539 PMC3948094 24493713

[25] Castellino RC, De Bortoli M, Lu X, Moon SH, Nguyen TA, Shepard MA, Rao PH, Donehower LA, Kim JY (2008). Medulloblastomas overexpress the p53-inactivating oncogene WIP1/PPM1D. J Neurooncol.

[26] Tabori U, Baskin B, Shago M, Alon N, Taylor MD, Ray PN, Bouffet E, Malkin D, Hawkins C (2010). Universal poor survival in children with medulloblastoma harboring somatic TP53 mutations. J Clin Oncol.

[27] de Bont JM, Packer RJ, Michiels EM, den Boer ML, Pieters R (2008). Biological background of pediatric medulloblastoma and ependymoma: a review from a translational research perspective. Neuro Oncol.

[28] Northcott PA, Korshunov A, Witt H, Hielscher T, Eberhart CG, Mack S, Bouffet E, Clifford SC, Hawkins CE, French P, Rutka JT, Pfister S, Taylor MD (2011). Medulloblastoma comprises four distinct molecular variants. J Clin Oncol.

[29] Taylor MD, Northcott PA, Korshunov A, Remke M, Cho YJ, Clifford SC, Eberhart CG, Parsons DW, Rutkowski S, Gajjar A, Ellison DW, Lichter P, Gilbertson RJ, Pomeroy SL, Kool M, Pfister SM (2011) Molecular subgroups of medulloblastoma: the current consensus. Acta Neuropathol doi:10.1007/s00401-011-0922-z 10.1007/s00401-011-0922-z PMC3306779 22134537

[30] Pei Y, Moore CE, Wang J, Tewari AK, Eroshkin A, Cho YJ, Witt H, Korshunov A, Read TA, Sun JL, Schmitt EM, Miller CR, Buckley AF, McLendon RE, Westbrook TF, Northcott PA, Taylor MD, Pfister SM, Febbo PG, Wechsler-Reya RJ (2012). An animal model of MYC-driven medulloblastoma. Cancer Cell.

[31] Kawauchi D, Robinson G, Uziel T, Gibson P, Rehg J, Gao C, Finkelstein D, Qu C, Pounds S, Ellison DW, Gilbertson RJ, Roussel MF (2012). A mouse model of the most aggressive subgroup of human medulloblastoma. Cancer Cell.

[32] Northcott PA, Shih DJ, Peacock J, Garzia L, Morrissy AS, Zichner T, Stutz AM, Korshunov A, Reimand J, Schumacher SE, Beroukhim R, Ellison DW, Marshall CR, Lionel AC, Mack S, Dubuc A, Yao Y, Ramaswamy V, Luu B, Rolider A, Cavalli FM, Wang X, Remke M, Wu X, Chiu RY, Chu A, Chuah E, Corbett RD, Hoad GR, Jackman SD (2012). Subgroup-specific structural variation across 1,000 medulloblastoma genomes. Nature.

[33] Robinson G, Parker M, Kranenburg TA, Lu C, Chen X, Ding L, Phoenix TN, Hedlund E, Wei L, Zhu X, Chalhoub N, Baker SJ, Huether R, Kriwacki R, Curley N, Thiruvenkatam R, Wang J, Wu G, Rusch M, Hong X, Becksfort J, Gupta P, Ma J, Easton J, Vadodaria B, Onar-Thomas A, Lin T, Li S, Pounds S, Paugh S (2012). Novel mutations target distinct subgroups of medulloblastoma. Nature.

[34] Pugh TJ, Weeraratne SD, Archer TC, Pomeranz Krummel DA, Auclair D, Bochicchio J, Carneiro MO, Carter SL, Cibulskis K, Erlich RL, Greulich H, Lawrence MS, Lennon NJ, McKenna A, Meldrim J, Ramos AH, Ross MG, Russ C, Shefler E, Sivachenko A, Sogoloff B, Stojanov P, Tamayo P, Mesirov JP, Amani V, Teider N, Sengupta S, Francois JP, Northcott PA, Taylor MD (2012). Medulloblastoma exome sequencing uncovers subtype-specific somatic mutations. Nature.

[35] Squire SE, Chan MD, Marcus KJ (2007). Atypical teratoid/rhabdoid tumor: the controversy behind radiation therapy. J Neurooncol.

[36] Grill J, Sainte-Rose C, Jouvet A, Gentet JC, Lejars O, Frappaz D, Doz F, Rialland X, Pichon F, Bertozzi AI, Chastagner P, Couanet D, Habrand JL, Raquin MA, Le Deley MC, Kalifa C (2005). Treatment of medulloblastoma with postoperative chemotherapy alone: an SFOP prospective trial in young children. Lancet Oncol.

[37] Jakacki RI, Feldman H, Jamison C, Boaz JC, Luerssen TG, Timmerman R (2004). A pilot study of preirradiation chemotherapy and 1800 cGy craniospinal irradiation in young children with medulloblastoma. Int J Radiat Oncol Biol Phys.

[38] (2012) Haplotype-based variant detection from short-read sequencing.http://arxiv.org/abs/1207.3907

[39] Wang K, Li M, Hakonarson H (2010). ANNOVAR: functional annotation of genetic variants from high-throughput sequencing data. Nucleic Acids Res.

[40] Abecasis GR, Auton A, Brooks LD, DePristo MA, Durbin RM, Handsaker RE, Kang HM, Marth GT, McVean GA (2012). An integrated map of genetic variation from 1,092 human genomes. Nature.

[41] Sherry ST, Ward MH, Kholodov M, Baker J, Phan L, Smigielski EM, Sirotkin K (2001). dbSNP: the NCBI database of genetic variation. Nucleic Acids Res.

[42] Do H, Wong SQ, Li J, Dobrovic A (2013). Reducing sequence artifacts in amplicon-based massively parallel sequencing of formalin-fixed paraffin-embedded DNA by enzymatic depletion of uracil-containing templates. Clin Chem.

[43] Robasky K, Lewis NE, Church GM (2014). The role of replicates for error mitigation in next-generation sequencing. Nat Rev Genet.

[44] Thorvaldsdottir H, Robinson JT, Mesirov JP (2013). Integrative Genomics Viewer (IGV): high-performance genomics data visualization and exploration. Brief Bioinform.

[45] Team RC (2013). R: A language and environment for statistical computing.

[46] Maechler M, Rousseeuw P, Struyf A, Hubert M, Hornik K (2013). cluster: Cluster Analysis Basics and Extensions. Therneau TMG, Patricia M (2013) A Package for Survival Analysis in S. R package version 2.37-4 edn.

[47] Hemming S, Cakouros D, Isenmann S, Cooper L, Menicanin D, Zannettino A, Gronthos S (2014). EZH2 and KDM6A act as an epigenetic switch to regulate mesenchymal stem cell lineage specification. Stem Cells.

[48] Lu X, Nguyen TA, Moon SH, Darlington Y, Sommer M, Donehower LA (2008). The type 2C phosphatase Wip1: an oncogenic regulator of tumor suppressor and DNA damage response pathways. Cancer Metastasis Rev.

[49] Takekawa M, Adachi M, Nakahata A, Nakayama I, Itoh F, Tsukuda H, Taya Y, Imai K (2000). p53-inducible wip1 phosphatase mediates a negative feedback regulation of p38 MAPK-p53 signaling in response to UV radiation. Embo J.

[50] Saldana-Meyer R, Recillas-Targa F (2011). Transcriptional and epigenetic regulation of the p53 tumor suppressor gene. Epigenetics.

[51] Riley T, Sontag E, Chen P, Levine A (2008). Transcriptional control of human p53-regulated genes. Nat Rev Mol Cell Biol.

[52] Courant F, Aksglaede L, Antignac JP, Monteau F, Sorensen K, Andersson AM, Skakkebaek NE, Juul A, Bizec BL (2010). Assessment of circulating sex steroid levels in prepubertal and pubertal boys and girls by a novel ultrasensitive gas chromatography-tandem mass spectrometry method. J Clin Endocrinol Metab.

[53] Fernandez-Cuesta L, Anaganti S, Hainaut P, Olivier M (2011). Estrogen levels act as a rheostat on p53 levels and modulate p53-dependent responses in breast cancer cell lines. Breast Cancer Res Treat.

[54] Yamamoto T, Mori T, Sawada M, Kuroboshi H, Tatsumi H, Yoshioka T, Matsushima H, Iwasaku K, Kitawaki J (2012). Estrogen-related receptor-gamma regulates estrogen receptor-alpha responsiveness in uterine endometrial cancer. Int J Gynecol Cancer.

[55] Parsons DW, Li M, Zhang X, Jones S, Leary RJ, Lin JC, Boca SM, Carter H, Samayoa J, Bettegowda C, Gallia GL, Jallo GI, Binder ZA, Nikolsky Y, Hartigan J, Smith DR, Gerhard DS, Fults DW, VandenBerg S, Berger MS, Marie SK, Shinjo SM, Clara C, Phillips PC, Minturn JE, Biegel JA, Judkins AR, Resnick AC, Storm PB, Curran T (2011). The genetic landscape of the childhood cancer medulloblastoma. Science.

[56] Hakimi AA, Chen YB, Wren J, Gonen M, Abdel-Wahab O, Heguy A, Liu H, Takeda S, Tickoo SK, Reuter VE, Voss MH, Motzer RJ, Coleman JA, Cheng EH, Russo P, Hsieh JJ (2013). Clinical and pathologic impact of select chromatin-modulating tumor suppressors in clear cell renal cell carcinoma. Eur Urol.

