# Supplementary material for: Mutation and expression analysis in medulloblastoma yields prognostic variants and a putative mechanism of disease for i17q tumors
Source: Acta Neuropathol Commun. 2014 Jul 17;2:74. doi: 10.1186/s40478-014-0074-1 (PMC4149211; doi:10.1186/s40478-014-0074-1)
Supplement: Additional file 4: Figure S1. — Expression of differentially-expressed chromatin remodeling genes in the four MB variants. Differential expression (mean values) was seen in histone deacetlyases, histone demethylases, histone methyltransferases, and other chromatin remodelers. These data suggest that, in general, group 3 and 4 tumors tend to increase histone methyltransferase activity while suppressing demethylase activity. Histone deacetylases vary tremendously by MB variant. Figure S2. Expression of chromatin remodeling genes differentially expressed across the four MB variants or previously associated with differential expression. Histone deacetylase genes HDAC1 and HDAC2 show differential expression across the MB variants, with decreased expression in groups D and C respectively. Histone methyltransferase-coding MLL2 is decreased in SHH MB, and is also frequently mutated in that group. EZH2 is involved in the H3K27 trimethylation, which is removed by KDM6A. Its gene EZH2 is relatively over-expressed in group 3 and 4 tumors. Previous studies had suggested that UTY (a paralog of KDM6A) and CDH7 may be decreased in expression in group 3 and 4 tumors in an effort to maintain an epigenetic stem-like state [40]. Significant differences for expression in these groups were not seen in this data set; however, this does not imply a stem-like epigenetic state is not maintained through other means in these tumors. Figure S3. Expression of chromatin remodeling genes whose mutations are over-represented in group 4/i17q in patients with and without i17q. Other than GPS2, there is no statistical significance (student t-test, 2-tailed) in expression of these genes between i17q-positive and negative tumors. The difference in GPS2 is likely explained by dosage effect as it is positioned in chromosome 17p. [file 40478_2014_74_MOESM4_ESM.pptx]

## Slide 1
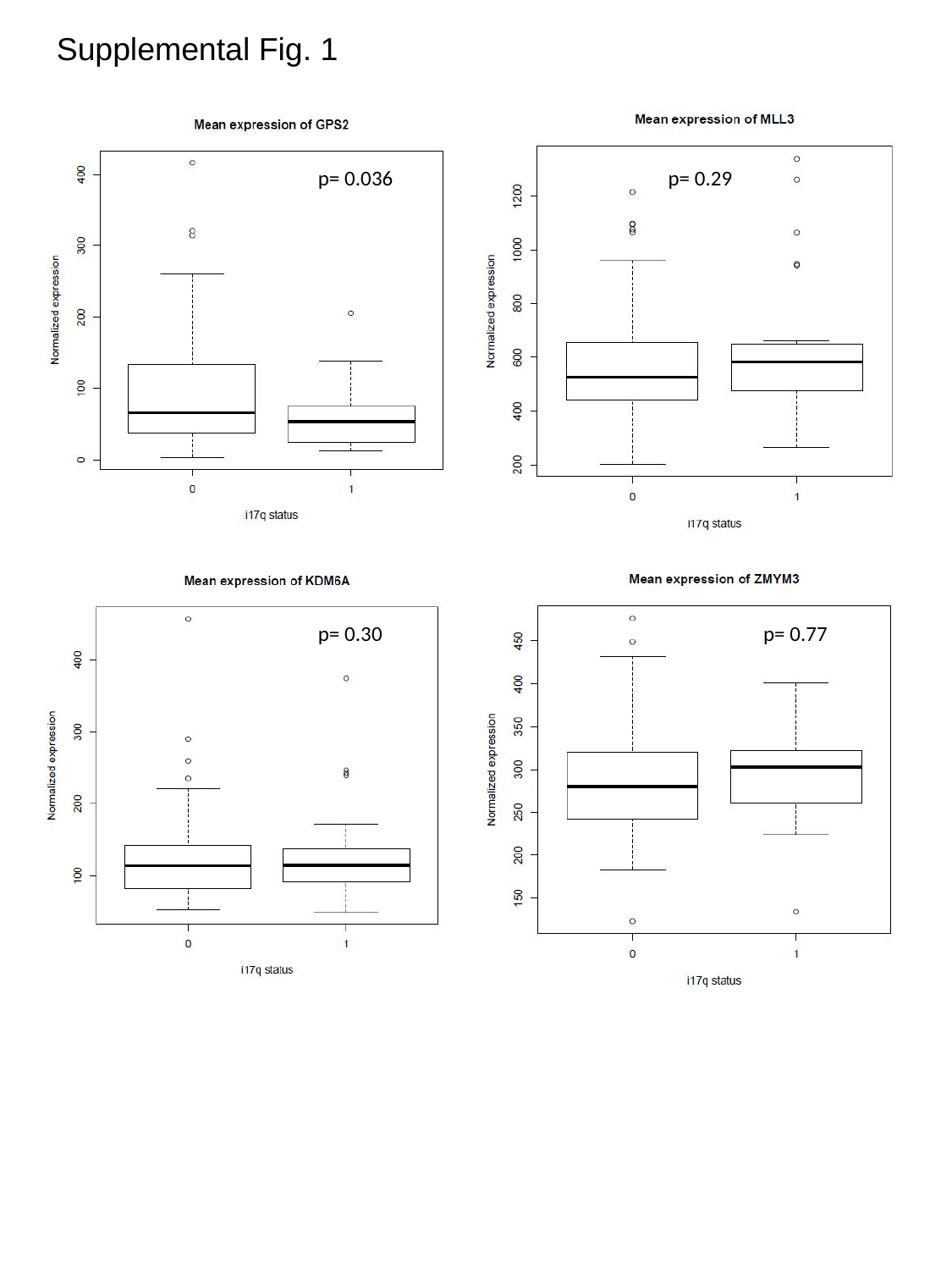

Supplemental Fig. 1
p= 0.036
p= 0.29
p= 0.30
p= 0.77

## Slide 2
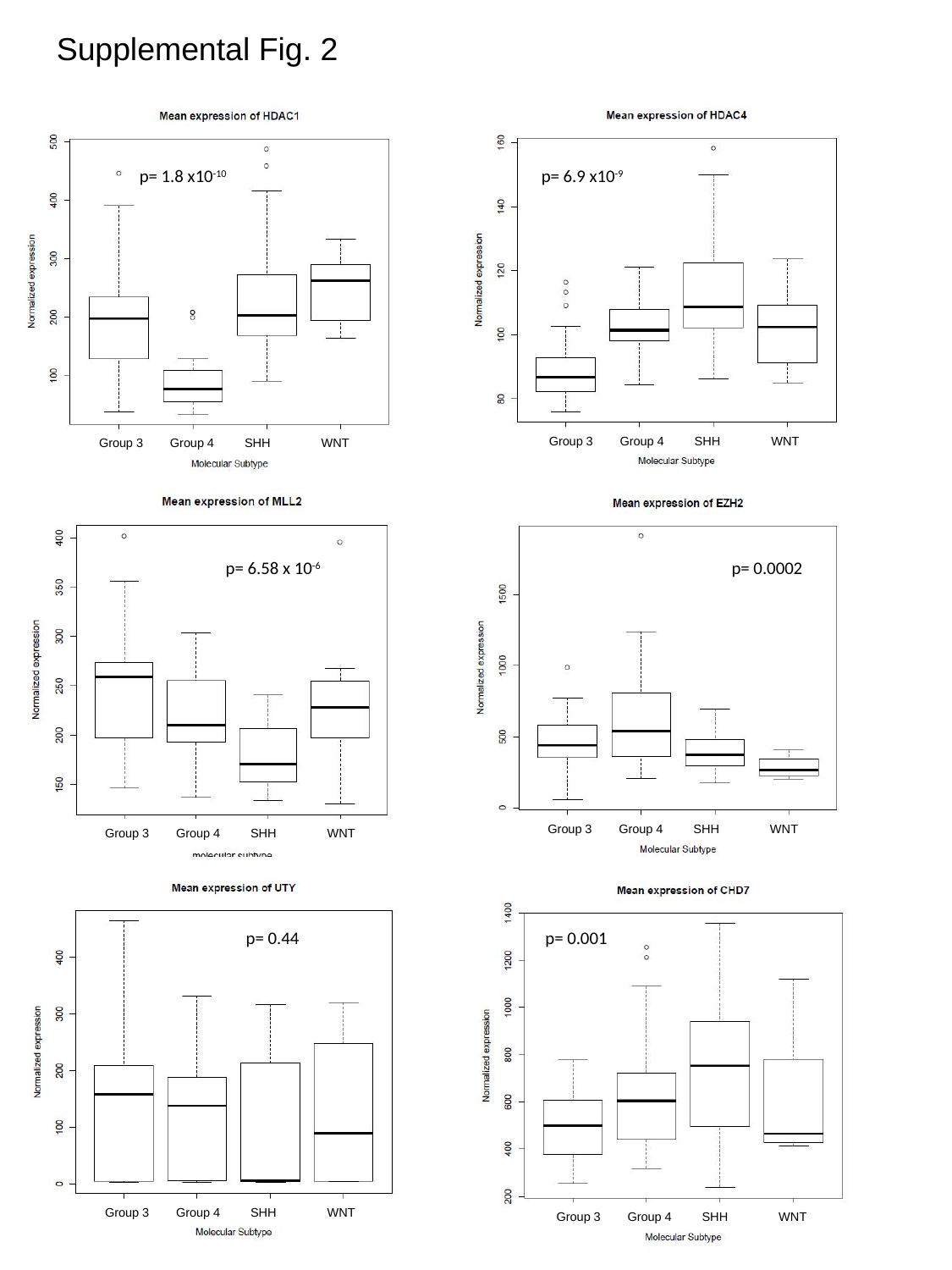

Supplemental Fig. 2
p= 1.8 x10^-10^
p= 6.9 x10^-9^
Group 3 Group 4 SHH WNT
Group 3 Group 4 SHH WNT
p= 6.58 x 10^-6^
p= 0.0002
Group 3 Group 4 SHH WNT
Group 3 Group 4 SHH WNT
p= 0.001
p= 0.44
Group 3 Group 4 SHH WNT
Group 3 Group 4 SHH WNT

## Slide 3
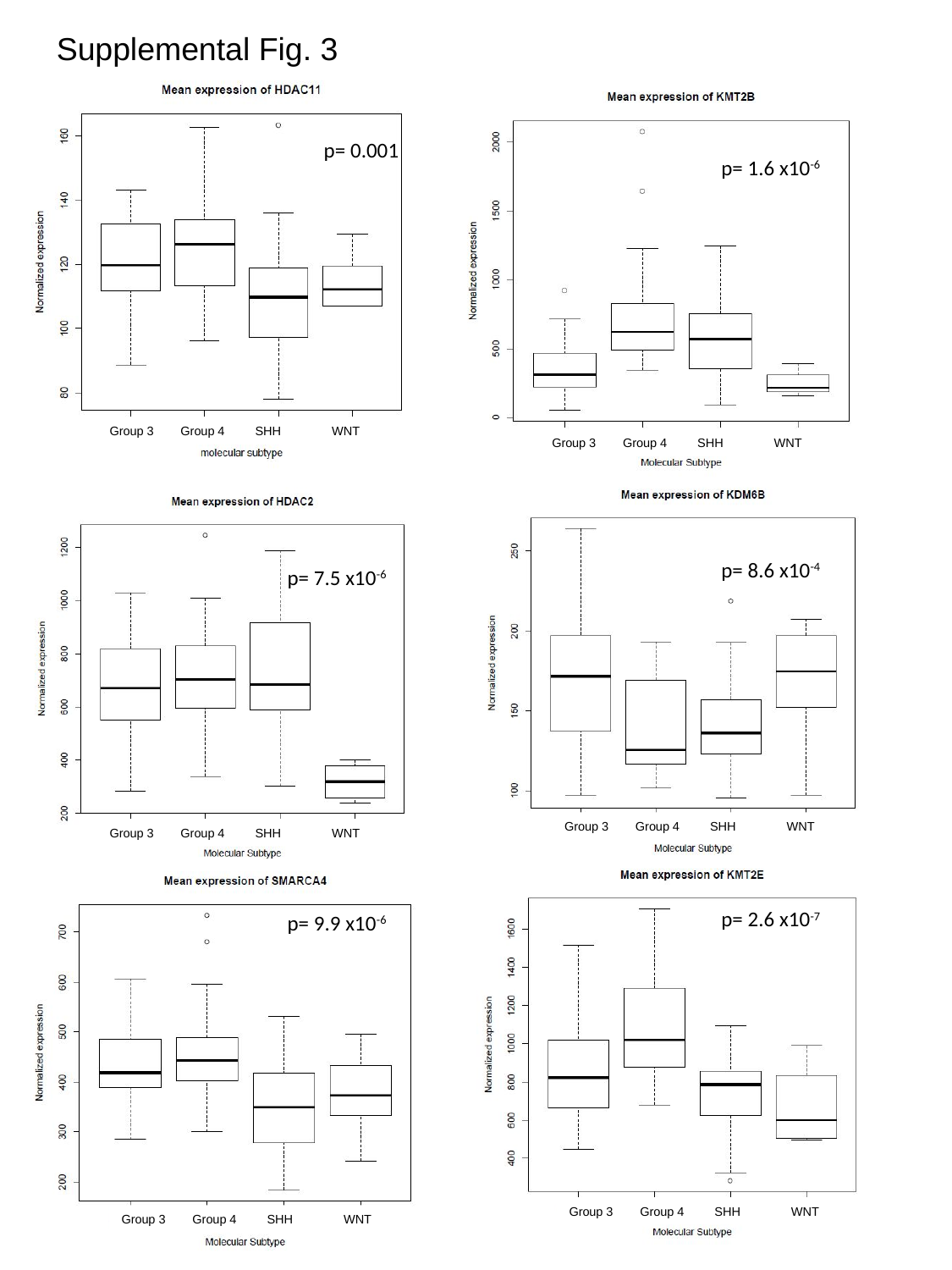

Supplemental Fig. 3
p= 0.001
p= 1.6 x10^-6^
Group 3 Group 4 SHH WNT
Group 3 Group 4 SHH WNT
p= 8.6 x10^-4^
p= 7.5 x10^-6^
Group 3 Group 4 SHH WNT
Group 3 Group 4 SHH WNT
p= 2.6 x10^-7^
p= 9.9 x10^-6^
Group 3 Group 4 SHH WNT
Group 3 Group 4 SHH WNT
