# Supplementary material for: Mutation and expression analysis in medulloblastoma yields prognostic variants and a putative mechanism of disease for i17q tumors
Source: Acta Neuropathol Commun. 2014 Jul 17;2:74. doi: 10.1186/s40478-014-0074-1 (PMC4149211; doi:10.1186/s40478-014-0074-1)
Supplement: Additional file 5: Figure S4. — Expression of DNA-methyltransferases is differentially expressed across MB variants. The SHH variant shows lower levels of expression for both DNMT1 and DNMT3A. Figure S5. Expression of TP53 and WIP1 in the molecular variants of MB. The top panel shows expression of TP53 in the MB molecular variants independent of any i17q-positive cases. Group 4 shows significantly lower expression, although it is not as low as seen in in i17q + tumors. The bottom figure shows that WIP1 is also differentially expressed among the MB variants, with significantly higher expression in Group 4. Figure S6. ESRRG expression in MB variants, i17q, and male and female sexes. ERRγ is an estrogen receptor that has no known ligand but can interfere with estradiol signaling. It has been shown than in ESRα-expressing cells, overexpression of ERRγ suppresses estradiol-mediated expression of its response elements. A. ESRRG codes for ERRγ and is significantly overexpressed in i17q tumors (p = 1.5 × 10^−5^, t-test). B. Similarly, group 4 tumors showed significantly higher levels compared to the other groups. C. Although male patients are significantly overrepresented in i17q-positive tumors and group 4 cases, male gender alone was not significantly associated with increased ESRRG expression, suggesting that this is intrinsic to group i17q/4 tumors. [file 40478_2014_74_MOESM5_ESM.pptx]

## Slide 1
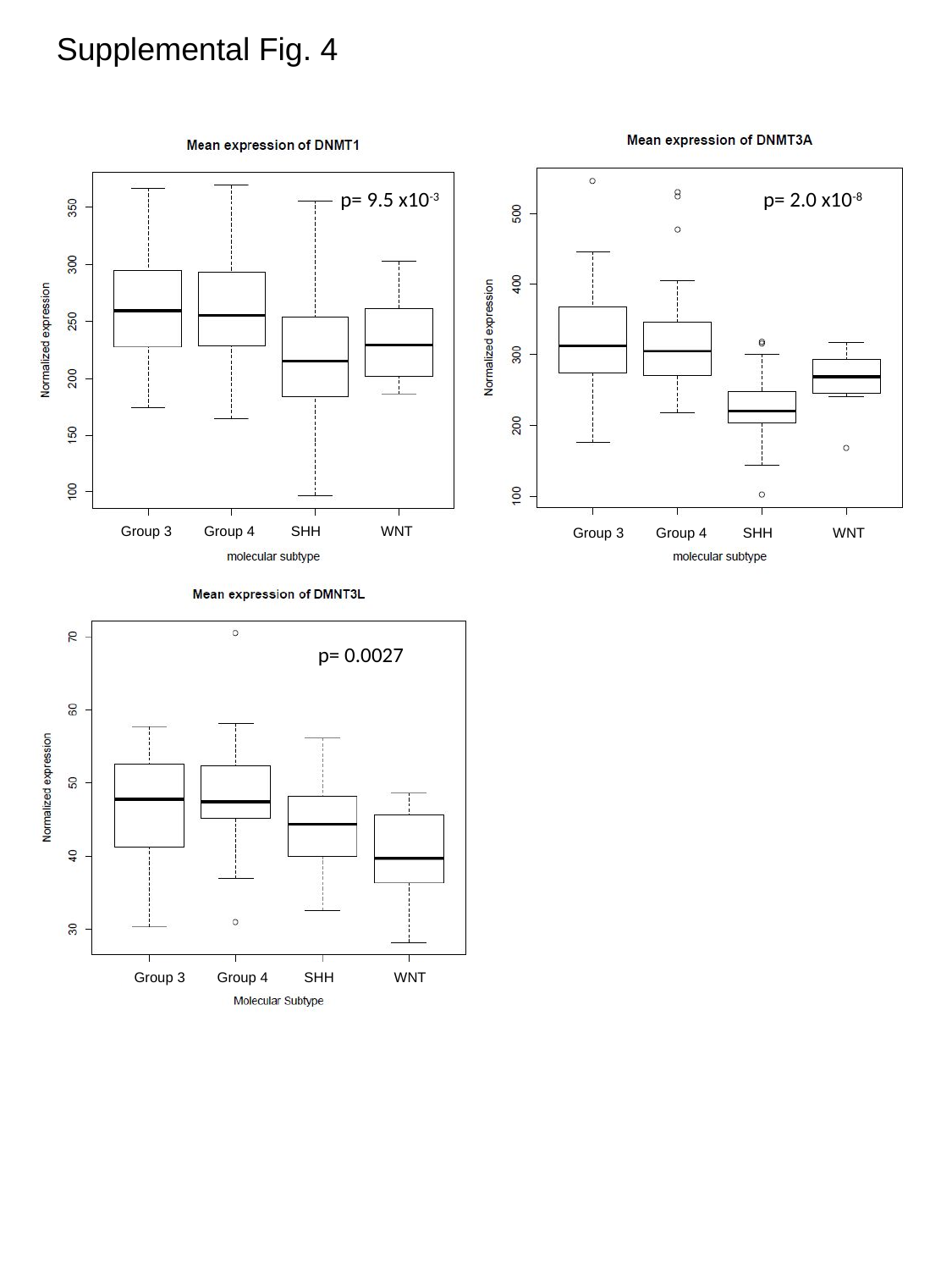

Supplemental Fig. 4
p= 9.5 x10^-3^
p= 2.0 x10^-8^
Group 3 Group 4 SHH WNT
Group 3 Group 4 SHH WNT
p= 0.0027
Group 3 Group 4 SHH WNT

## Slide 2
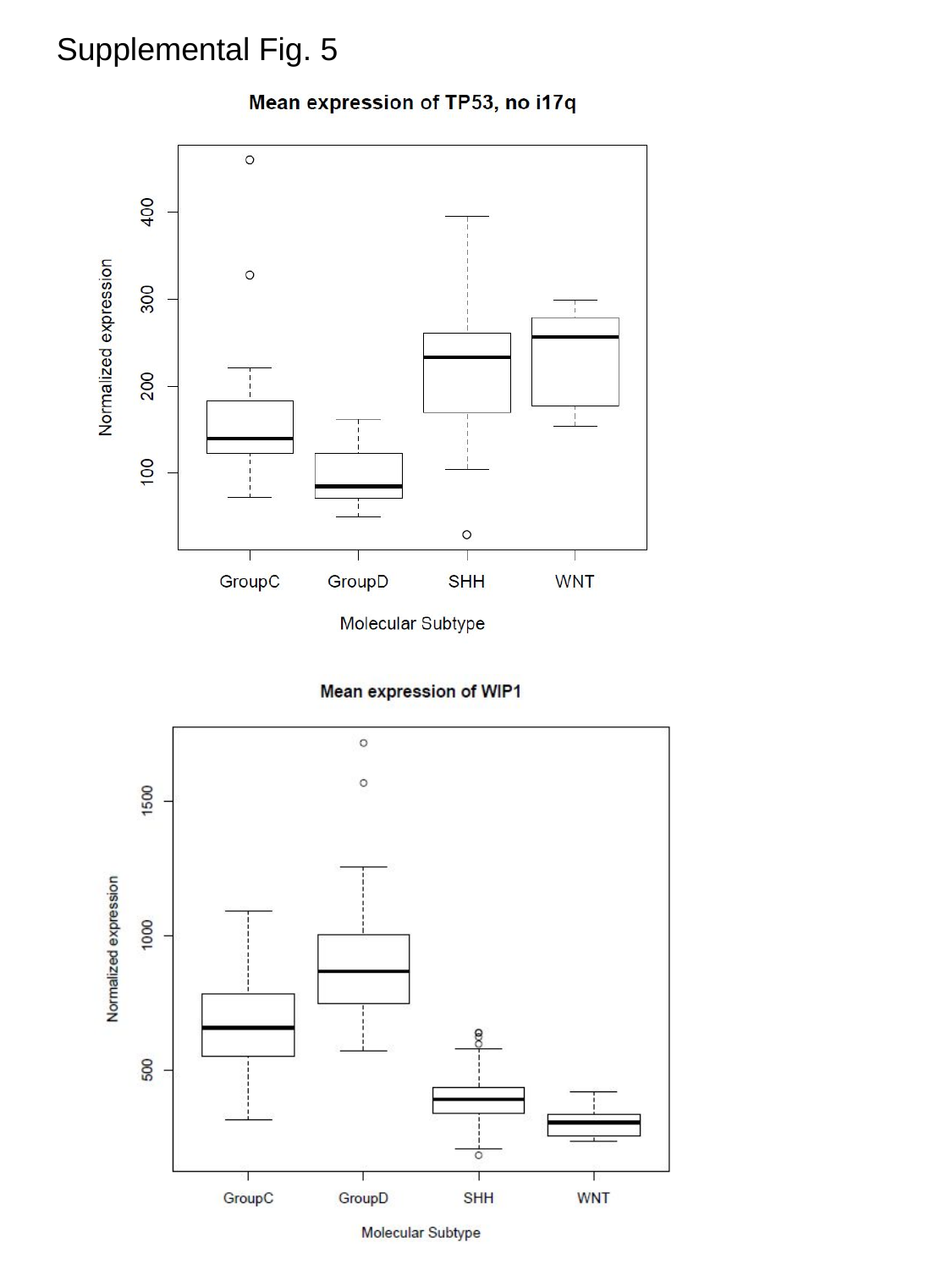

Supplemental Fig. 5

## Slide 3
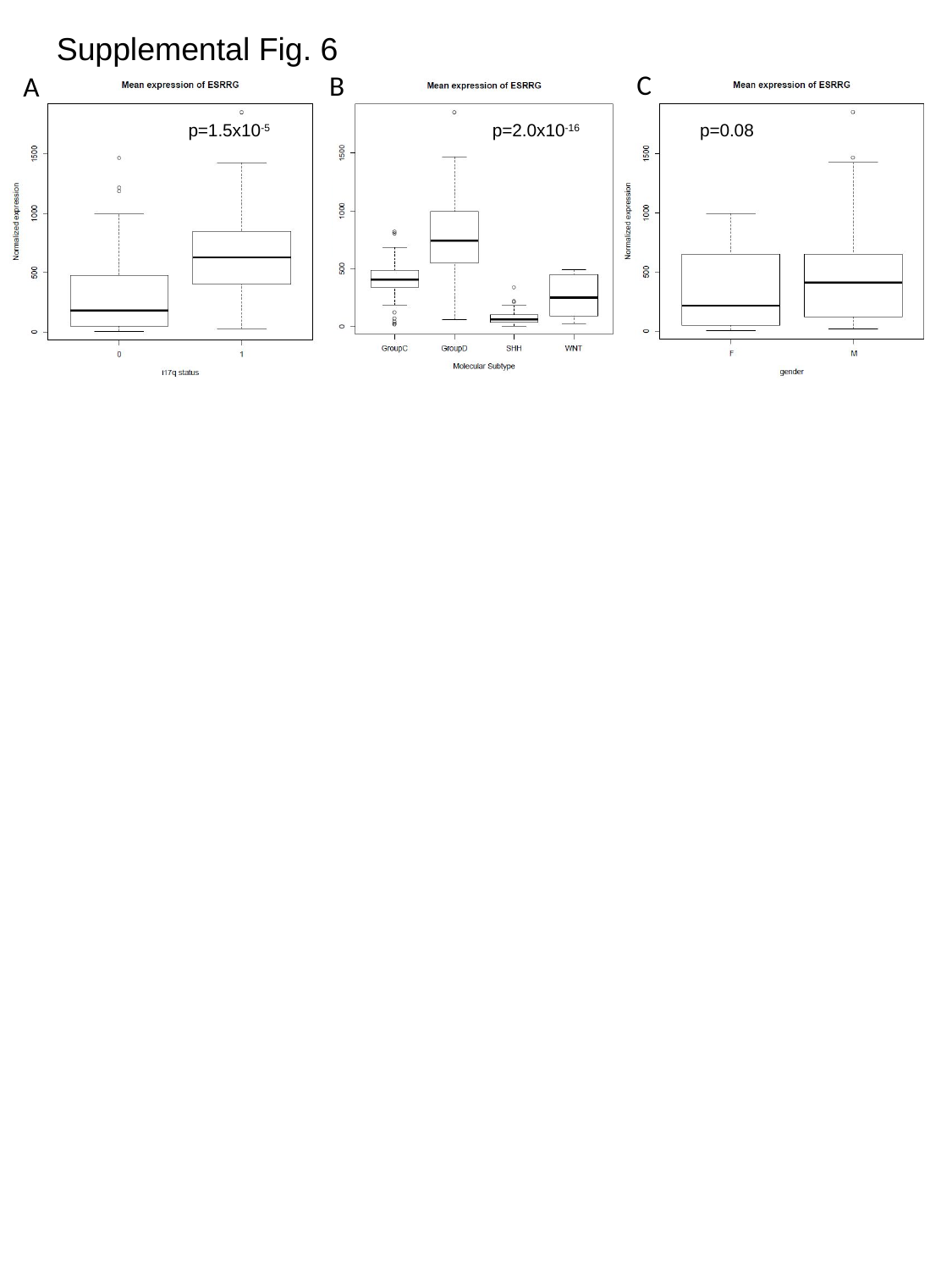

Supplemental Fig. 6
C
B
A
p=2.0x10^-16^
p=1.5x10^-5^
p=0.08
